# Supplementary figures and images for: Evaluation of DNA extraction protocols from liquid-based cytology specimens for studying cervical microbiota
Source: PLoS One. 2021 Aug 30;16(8):e0237556. doi: 10.1371/journal.pone.0237556 (PMC8404996; doi:10.1371/journal.pone.0237556)

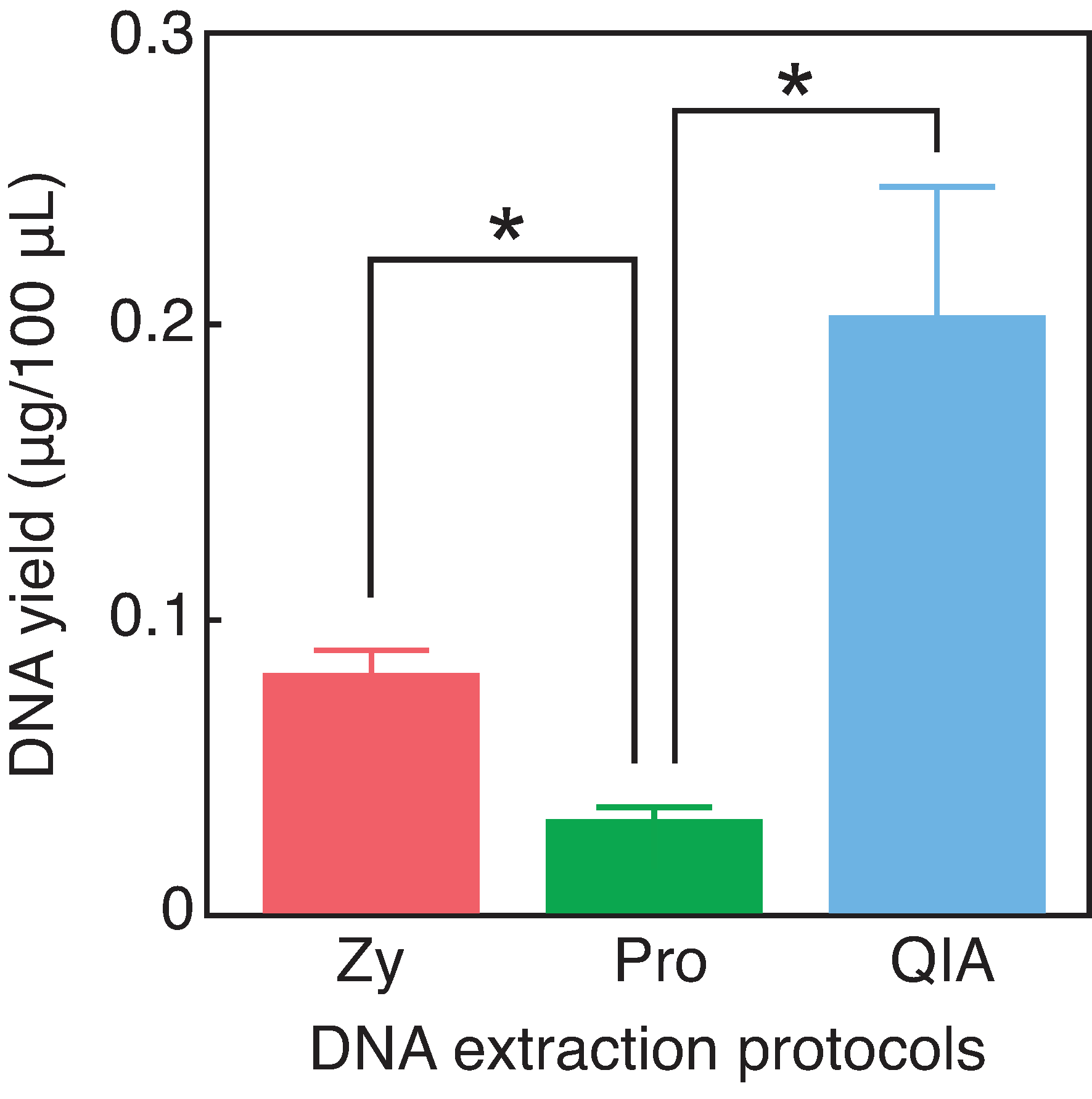

Supplement: S1 Fig — DNA yield of QIAampMini was significantly higher than that of PowerFecalPro (p < 0.001, Dunn’s test with Benjamini-Hochberg-adjustment). Also, the DNA yield of ZymoBIOMICS was significantly higher than that of PowerFecalPro (p < 0.001, Dunn’s test with Benjamini-Hochberg-adjustment). The amount of DNA was calculated based on the absorbance of nucleic acids measured by Nanodrop One. By the protocol recommended by the manufacturer, nucleic acid (Poly-A carrier) was used in IndiSpin. Therefore, IndiSpin was excluded from the analysis of DNA yield. The amount of DNA yield per 100 μL ThinPrep sample volume were compared. The bar graph shows the mean and standard deviation. Zy: ZymoBIOMICS DNA Miniprep Kit, Pro: QIAamp PowerFecal Pro DNA Kit, QIA: QIAamp DNA Mini Kit. (TIF) [file pone.0237556.s001.tif]

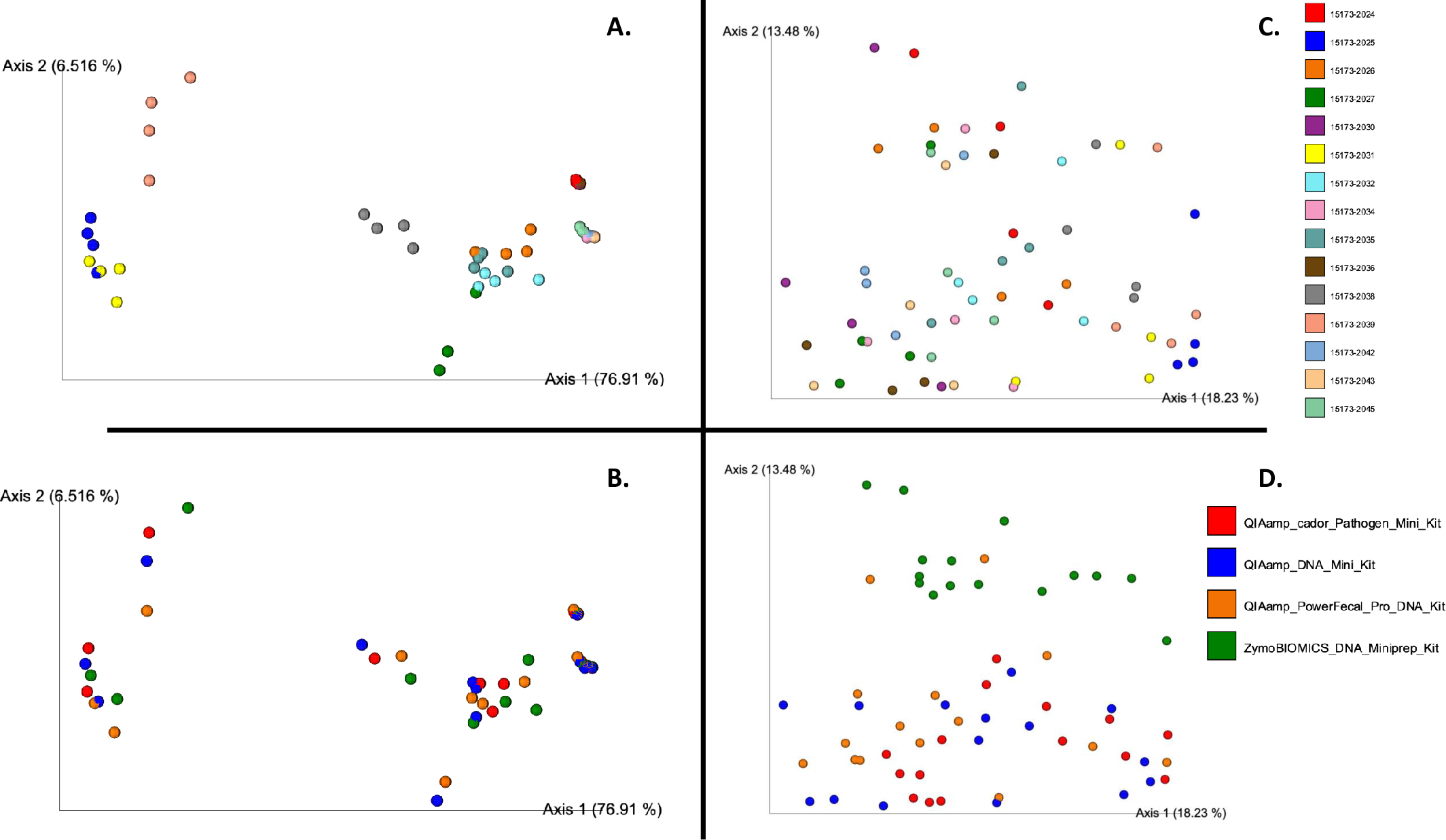

Supplement: S2 Fig — Weighted UniFrac (A & B) and Unweighted UniFrac (C & D), PCoA colored by subject ID (top row) and DNA extraction kit (bottom row). Weighted UniFrac clusters samples by subject whereas Unweighted UniFrac appears more sensitive to the type of DNA extraction kit. Data were rarefied to 51,197 reads per sample. (TIF) [file pone.0237556.s002.tif]

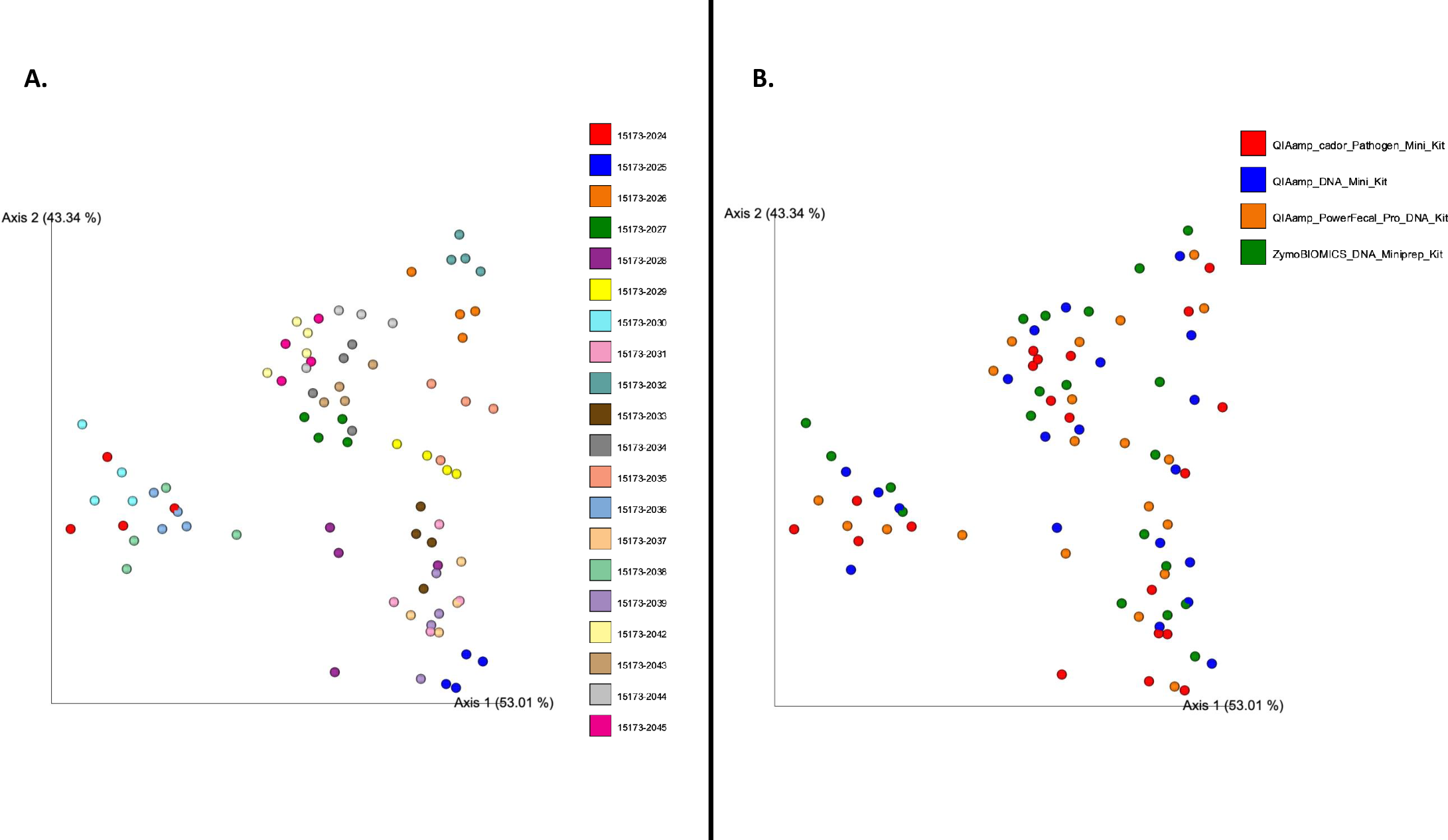

Supplement: S3 Fig — Non-rarefaction-based analysis of beta-diversity. Samples are colored by individual subject ID (A) and DNA extraction kit (B). Samples predominately cluster by subject and not DNA extraction kit. (TIF) [file pone.0237556.s003.tif]

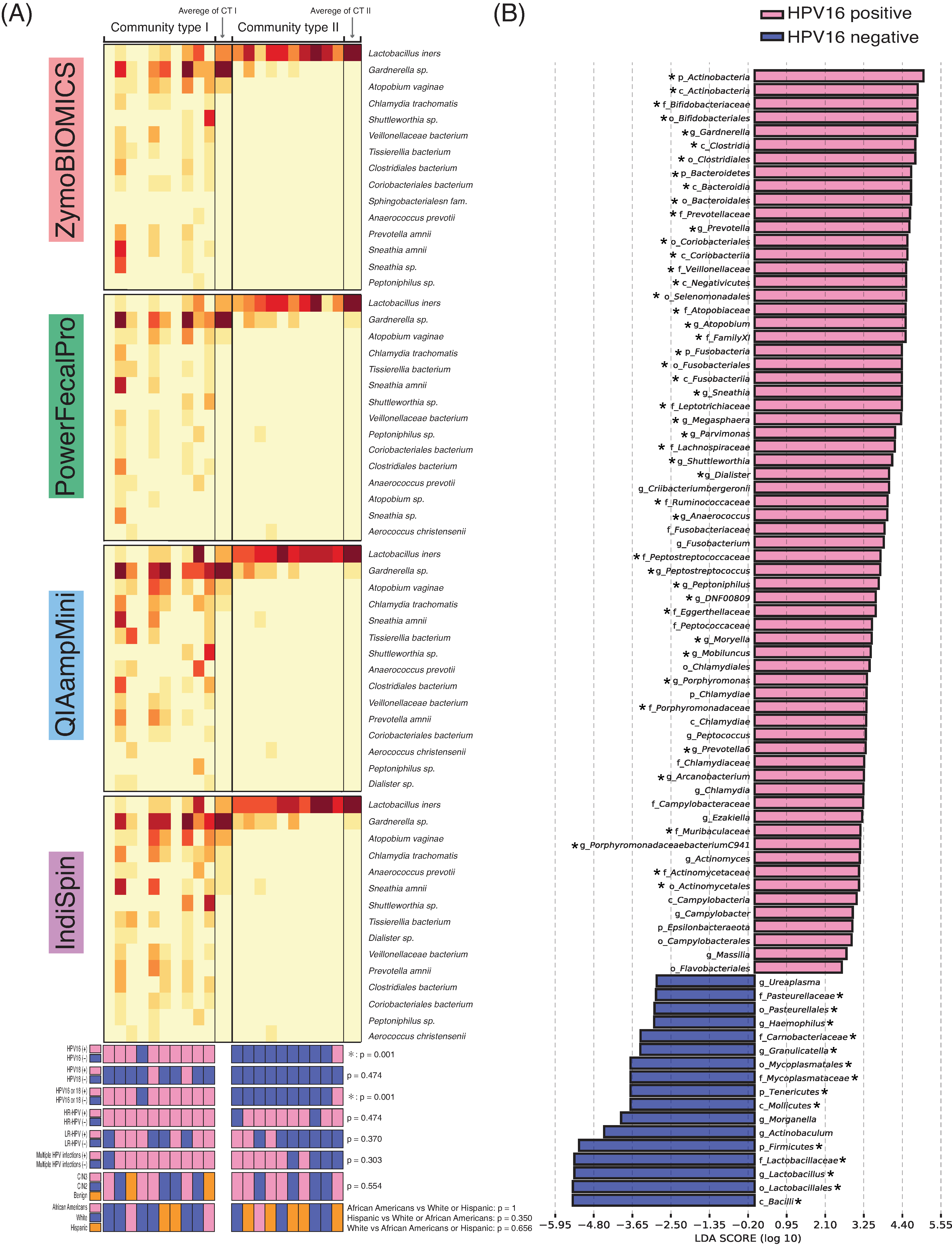

Supplement: S4 Fig — Community type and HPV 16 assessed by using 4 kits (A) Community types were classified into two types in all DNA extraction kits, mainly based on the percentage of Lactobacillus iners. HPV16 infection was negatively associated with the dominance of L. iners (community type I; p = 0.001, Fisher’s exact test) regardless of DNA extraction method. Although, we observed slight variation in the abundance of microbiota across the extraction kits (even within the same individual patient), the ability to detect two community types was identical across all DNA extraction kits. No significant differences were observed in the relationship of other phenotypes of patients (HPV18, HR-HPV, LR-HPV, multiple HPV infections, Biopsy, and Race). The top 15 bacteria detected for each DNA extraction kit are shown. Samples were clustered by the Dirichlet component. Narrow columns show each sample and a broader column shows averages of samples. Rows show taxa at the species level. Dark or thin colors correspond to larger or smaller counts of OTUs, respectively. CT: community type. (B) LEfSe analysis, using combined data from all four kits detected a significant enrichment of 66 taxa in the cervical environment with HPV16 infection and 17 taxa without HPV16 infection. Genus Lactobacillus were enriched in the HPV16 negative patients (p < 0.001, LDA score: 5.38). Asterisks denote taxa that were significant after post-hoc significant testing with Scheffe test [64]. (TIF) [file pone.0237556.s004.tif]
